# Microfluidics co-culture systems for studying tooth innervation

**DOI:** 10.3389/fphys.2014.00326

**Published:** 2014-08-25

**Authors:** Pierfrancesco Pagella, Estrela Neto, Lucia Jiménez-Rojo, Meriem Lamghari, Thimios A. Mitsiadis

**Affiliations:** ^1^Department of Orofacial Development and Regeneration, Faculty of Medicine, Centre for Dental Medicine, Institute of Oral Biology, University of ZurichZurich, Switzerland; ^2^NEW Therapies Group, INEB - Instituto de Engenharia Biomédica, Universidade do PortoPorto, Portugal; ^3^Faculdade de Medicina da Universidade do PortoPorto, Portugal; ^4^Instituto de Ciências Biomédicas Abel Salazar, Universidade do PortoPorto, Portugal

**Keywords:** microfluidics, dental mesenchyme, tooth development, innervation, mineralization, orofacial, trigeminal ganglia

## Abstract

Innervation plays a key role in the development and homeostasis of organs and tissues of the orofacial complex. Among these structures, teeth are peculiar organs as they are not innervated until later stages of development. Furthermore, the implication of neurons in tooth initiation, morphogenesis and differentiation is still controversial. Co-cultures constitute a valuable method to investigate and manipulate the interactions of nerve fibers with their target organs in a controlled and isolated environment. Conventional co-cultures between neurons and their target tissues have already been performed, but these cultures do not offer optimal conditions that are closely mimicking the *in vivo* situation. Indeed, specific cell populations require different culture media in order to preserve their physiological properties. In this study we evaluate the usefulness of a microfluidics system for co-culturing mouse trigeminal ganglia and developing teeth. This device allows the application of specific media for the appropriate development of both neuronal and dental tissues. The results show that mouse trigeminal ganglia and teeth survive for long culture periods in this microfluidics system, and that teeth maintain the attractive or repulsive effect on trigeminal neurites that has been observed *in vivo*. Neurites are repealed when co-cultured with embryonic tooth germs, while postnatal teeth exert an attractive effect to trigeminal ganglia-derived neurons. In conclusion, microfluidics system devices provide a valuable tool for studying the behavior of neurons during the development of orofacial tissues and organs, faithfully imitating the *in vivo* situation.

## Introduction

Innervation plays a key role in both development and homeostasis of organs and tissues (Kumar and Brockes, 2012; Pagella et al., 2014). Increasing evidence supports the notion that innervation is also involved in the regulation of stem cell proliferation, mobilization and differentiation (Katayama et al., 2006; Brownell et al., 2011; Fitch et al., 2012). For example, recent studies have shown that in the orofacial complex parasympathetic nerves are necessary for epithelial progenitor cells function during the development of salivary glands, as well as for their regeneration during adult life (Knox et al., 2010, 2013). Similarly, it has been demonstrated that innervation is necessary for the development and maintenance of taste buds (Oakley et al., 1998; Mistretta et al., 1999; Sun and Oakley, 2002; Oakley and Witt, 2004). In this context, it is important to better understand the role of sensory innervation during odontogenesis.

Teeth are peculiar organs since their innervation starts shortly after embryonic development. Teeth develop as a result of sequential and reciprocal interactions between the oral ectoderm and cranial neural crest-derived mesenchyme. These interactions give rise to epithelial-derived ameloblasts and mesenchyme-derived odontoblasts that are responsible for the formation of enamel and dentin, respectively (Thesleff et al., 1995; Mitsiadis and Graf, 2009). Sensory nerves from the trigeminal ganglia and sympathetic nerves from the superior cervical ganglia innervate the adult teeth (Mohamed and Atkinson, 1983; Johnsen, 1985; Luukko, 1997). During embryogenesis, nerve fibers emanating from the trigeminal ganglia project toward the developing tooth germs and progressively surround them but they do not penetrate into the dental papilla (Mohamed and Atkinson, 1983). Nerve fibers enter the dental pulp at more advanced developmental stages that coincide with odontoblast differentiation and dentin matrix deposition (Mitsiadis et al., 1992). Dental pulp innervation is completed soon after tooth eruption in the oral cavity (Mohamed and Atkinson, 1983). Although it has been clearly demonstrated that innervation is a prerequisite for tooth formation in fishes (Tuisku and Hildebrand, 1994), the role of innervation in tooth initiation and development still remains controversial in mammals (Kollar and Lumsend, 1979; Lumsend and Buchanan, 1986; Løes et al., 2002). Previous studies have revealed that semaphorins and neurotrophins are involved in the regulation of innervation during odontogenesis (Mitsiadis et al., 1992; Mitsiadis and Luukko, 1995; Kettunen et al., 2005; Moe et al., 2012). Recently, it has been demonstrated that sensory nerves regulate mesenchymal stem cell homeostasis in mouse incisors via secretion of Sonic Hedgehog (Shh) (Zhao et al., 2014). However, there is still not sufficient information about the role of innervation in the development and repair of dental tissues.

Co-cultures constitute a valuable method to investigate and manipulate the interactions between nerve fibers and teeth in a controlled and isolated environment (Lumsend and Davies, 1986; Lillesaar and Fried, 2004). At the same time, co-culturing is subject to various technical adjustments. For example, nerves and specific dental tissues (e.g., pulp, dental follicle, enamel epithelium) often require different culture media in order to survive for long periods of time (Mitsiadis and Drouin, 2008; Petrinovic et al., 2010; Otsu et al., 2012).

Conventional co-cultures have been used during the last decades to investigate the attractive effects of oral and dental tissues from different developmental stages on sensory nerve fibers (Lumsend and Davies, 1986; Lillesaar et al., 2001; Lillesaar and Fried, 2004). For that purpose, cultures of trigeminal ganglia and their target tissues were performed in the same culture medium for very short periods (i.e., 2 days). However, extension of the culture period is required to investigate the effects of innervation on tooth morphogenesis and differentiation. For this reason, non-contiguous co-cultures would be more suitable to perform studies on neuronal-dental tissue interactions.

Microfluidics devices allow co-cultures of neurons and different dental cell types or entire tooth germs in their appropriate culture media. In these devices, dental tissues and neurons are separated in different compartments, while allowing the growth of axons from the neural cell bodies through microgrooves toward the compartment containing their target tissue (Park et al., 2006). Microfluidics co-culture devices have been already used to study the interactions between neurons and microglia (Hosmane et al., 2012; Delamarche et al., 2013), as well as cell to cell interactions in cancer and neovascularization (Delamarche et al., 2013).

Aim of this study is to evaluate the usefulness of a microfluidics system for co-culturing mouse trigeminal ganglia and tooth germs from different developmental stages. We tested whether trigeminal ganglia and teeth are able to survive for long periods of time when co-cultured in microfluidic devices. Moreover, we investigated whether teeth from different developmental stages maintain in these *in vitro* conditions the same repulsive or attractive effects on trigeminal innervation.

## Materials and methods

### Animals and tissues processing

C57/BL6 mice were used at embryonic (E) and postnatal (PN) stages. Embryonic age was determined according to vaginal plug (day 0.5) and confirmed by morphological criteria. All mice were maintained and handled according to the Swiss Animal Welfare Law and in compliance with the regulations of the Cantonal Veterinary Office, Zurich.

Trigeminal ganglia were dissected from embryonic day 15.5–16.5 (E15.5–E16.5) mouse embryos. Incisor tooth germs were dissected from E15.5 embryos, while molar tooth germs were obtained from E17.5 embryos and PN5 pups. Dissections were performed in Dulbecco's phosphate buffered saline (PBS).

### Antibodies and proteins

The following primary antibodies were used: mouse IgG1 Neurofilament antibody (Hybridoma Bank, Iowa City, IA, USA) diluted 1:100 in 1% bovine albumin serum (BSA)/PBS, mouse IgG1 β-Tubulin III antibody (Sigma-Aldrich, Switzerland) diluted 1:1000 in 1% BSA/PBS. The following secondary antibodies were used: Alexa-Fluor488 conjugated α-Mouse, Alexa-fluor568 conjugated α-Rabbit (Invitrogen–Life Technologies, Switzerland) diluted 1:500 in 1% BSA/PBS, HRP-conjugated Goat α-Mouse-IgG1 (Southern Biotech, Switzerland) diluted 1:2000 in 1% BSA/PBS.

Nerve growth factor (recombinant rat beta NGF, 556-NG) protein (R&D Systems, Minneapolis, MN, USA) was added to the culture medium for trigeminal ganglia at a final concentration of 50 ng/ml.

### Culture media for organotypic cultures of trigeminal ganglia and tooth germs

Trigeminal ganglia were cultured in a Neurobasal medium (Gibco) supplemented with B27 (Gibco 17504-044), L-glutamine, 1% penicillin/streptomycin and 50 ng/ml NGF. Molars were cultured in a medium containing DMEM (high glucose 4.5 mg/ml) (GE Healthcare, UK), 20% Fetal Bovine Serum (FBS) (Pansera, Germany), L-Glutamine, 1% penicillin/streptomycin and 0.9 mM ascorbic acid.

### Conventional co-cultures of embryonic trigeminal ganglia (TG) and tooth germs

Round glass coverslips were placed in 4-well-plates and coated with 0.1 mg/ml poly-D-lysine overnight and thereafter with laminin (5 μg/ml) (Sigma-Aldrich, Switzerland), in Neurobasal medium (Gibco—Life Technologies, Switzerland) for 2 h at 37°C. Laminin solution was substituted with a medium specific for the growth of trigeminal ganglia as described above. After dissociation trigeminal ganglia and incisors were placed on the poly-D-lysine/laminin coated coverslips and co-cultured in the medium previously described.

### Co-cultures in microfluidic devices

Microfluidic devices (AX150, AX450, Millipore, Switzerland) were punched with a 1 mm diameter biopsy punch. Devices were assembled and coated with 0.1 mg/ml poly-D-lysine and 5 μg/ml laminin as previously described (Neto et al., 2014). Trigeminal ganglia were placed in the co-culture platform immediately after dissection. Co-cultures were performed at 37°C in a 5% CO_2_ incubator. Molars were added to the co-culture systems 3 days after trigeminal ganglia placement. Thereafter, co-cultures were maintained for 10 days with daily medium change. After culture, samples were washed with PBS and fixed with 4% paraformaldehyde (PFA) for 15 min.

## Immunohistochemisty

### Immunofluorescence

Neurites were detected using antibodies against neurofilament and β-Tubulin III. Alexa-fluor 488 or 568 anti-mouse secondary antibodies were used. Samples were then stained with DAPI (4′ 6-diamidino-2-phenylindole) and analyzed with the Leica DM6000 FS microscope. Pictures were taken using the Leica DFC350FX camera and the Leica Application Suite Advanced Fluorescence (LAS AF) software.

### Immunohistochemistry

Teeth were removed from the microfluidic devices, embedded in Tissue Tek® (Sakura) and sectioned (thickness: 20 μm). Neurites were detected using a mouse monoclonal antibody against neurofilament. Immunoperoxidase (ABC kit, Vector Laboratories, Switzerland) staining was performed as previously described (Mitsiadis et al., 1992, 1993).Omission of the primary antibody served as a negative control. Samples were counterstained with toluidine blue. Pictures were taken using the Leica DFC420C camera and the Leica Application Suite (LAS) software.

## Results

### Conventional co-cultures of embryonic trigeminal ganglia and tooth germs

**Figure 1:**
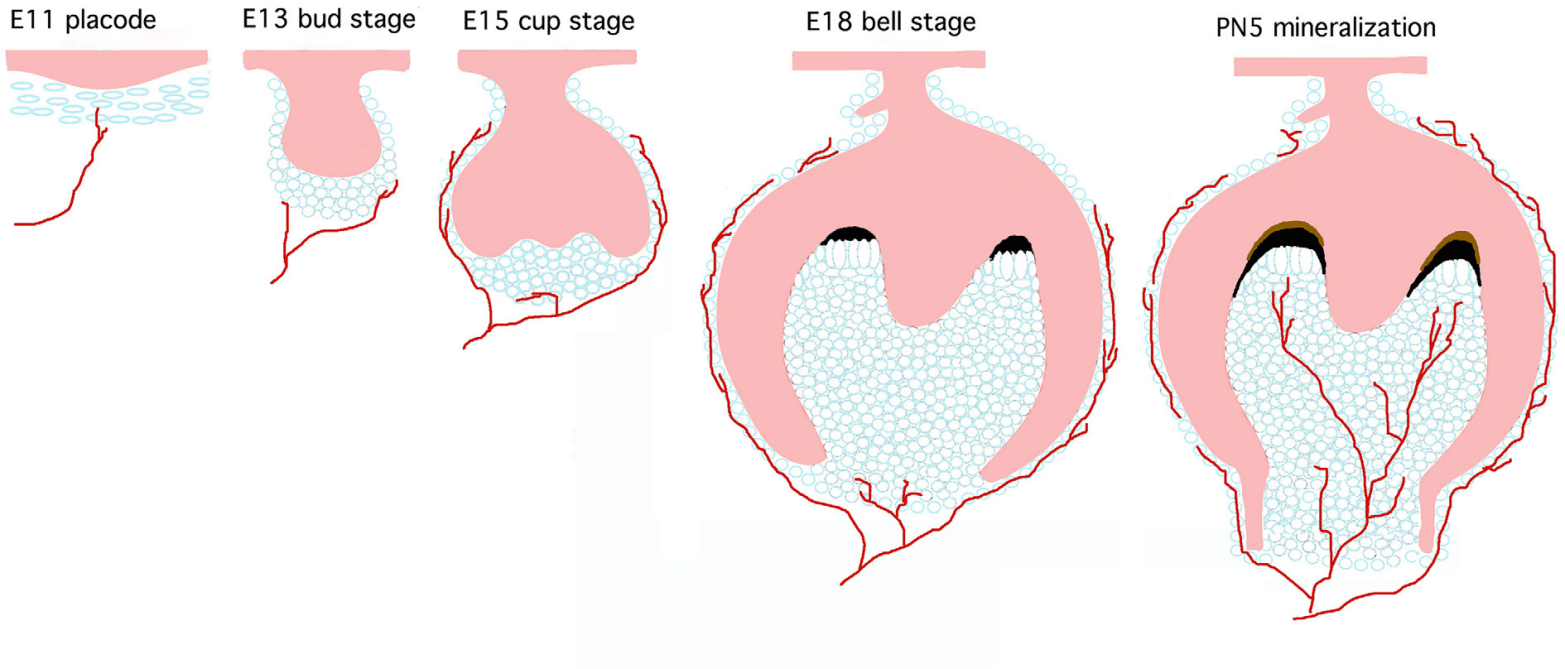
**Mouse tooth development and innervation**. Teeth develop through continuous interactions between dental epithelium (pink) and neural crest-derived mesenchyme (light blue). Axons from the trigeminal nerve project toward the tooth germ from the earliest developmental stages and surround the developing tooth germs. Nerves penetrate into the dental pulp mesenchyme only at later stages of development.

**Figure 2:**
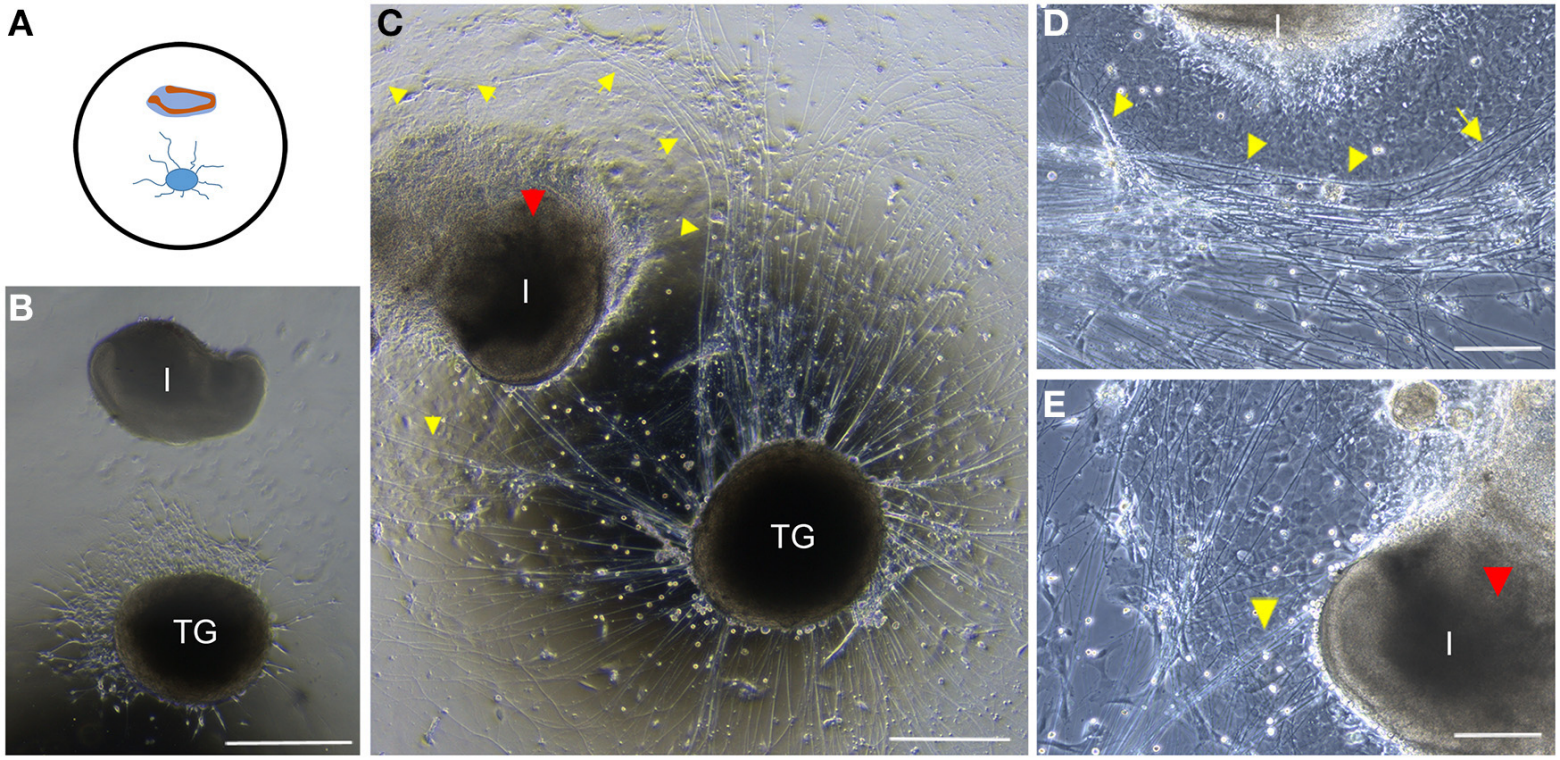
**Conventional co-cultures of trigeminal ganglia (TG) and incisors. (A)** Schematic representation of the disposition of embryonic trigeminal ganglion and incisor in co-culture on glass dishes. **(B)** Trigeminal ganglion (TG) and incisor (I) after 4 days in co-culture. **(C)** Trigeminal ganglion and incisor after 10 days in co-culture. Axons (yellow arrowheads) are clearly repealed by the incisor. The incisor presents signs of degeneration that are evident (red arrowhead) **(D)** Neurites grow through the layer of cells surrounding the incisor germ (yellow arrowheads). **(E)** Few neurites grow toward the labial cervical loop (yellow arrowhead). Signs of tooth degeneration are clear (red arrowhead). Scale bars: 500 μm for **(B,C)** and 200 μm for **(D,E)**. Abbreviations: TG, trigeminal ganglion; I, incisor.

During tooth development, axons projecting from trigeminal nerves approach and innervate the dental follicle mesenchyme, but they do not enter the dental papilla mesenchyme until later stages of development (Figure 1). In order to investigate neuronal-dental tissue interactions we performed conventional co-cultures of E15.5 mouse embryonic trigeminal ganglia and incisor germs (Figures 2A,B). Cultures were performed in a medium optimized for trigeminal ganglia survival and growth. During the first 4 days of culture neurites were grown from the ganglia and extended in all directions (Figure 2B). A clear repeal effect was observed once the growing neurites approached the incisor tooth germs (Figure 2C). Neurites grew around the incisors on top of a layer of mesenchymal cells surrounding the incisor (Figures 2C,D). However, most of the axons did not approach and never penetrated into the tooth germs during the whole culture period (Figure 2C). Only few axons have reached the posterior part (cervical loop area) of the incisor (Figure 2E). In these culture conditions the incisor germ did not grow properly and signs of dental tissue degeneration were clearly observable after long periods of culture (Figures 2C,E); teeth did not grow and lost their structure (red arrowhead, Figures 2C,E, see Supplementary Figure 1).

### Co-cultures of embryonic trigeminal ganglia and embryonic or postnatal tooth germs in microfluidic devices

**Figure 3:**
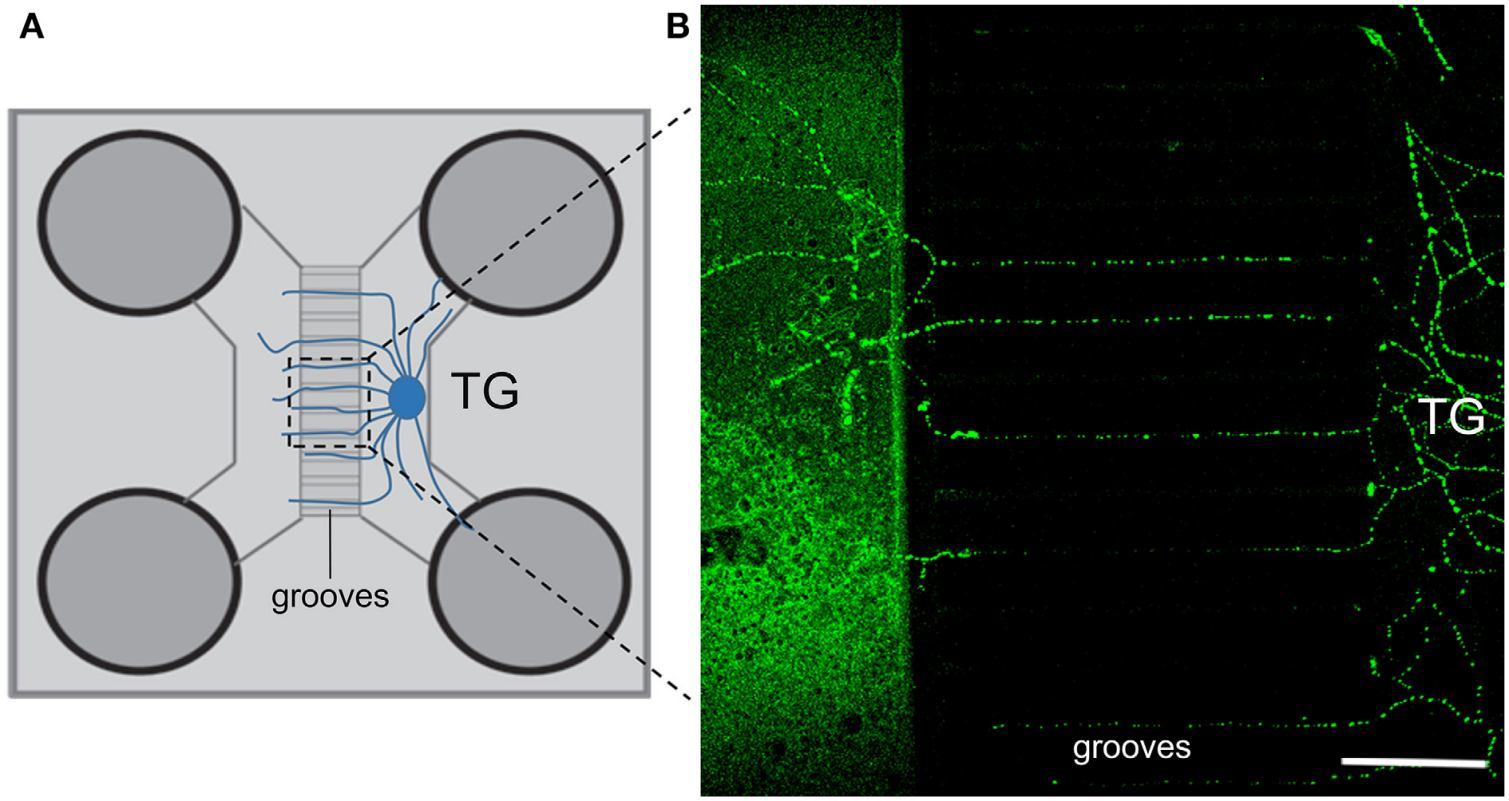
**General setup and growth of neurites through the microgrooves of the microfluidics device**. **(A)** Schematic view of a microfluidics co-culture system device. **(B)** Both β-tubulin (green) and neurofilament (not shown) immunostaining show extensive neurite outgrowth toward the empty compartment of the device after 4 days of culture. Scale bars: 150 μm. TG, trigeminal ganglion.

In order to grow both trigeminal ganglia and teeth in optimal culture conditions we used microfluidics devices. This system allows the co-culturing of ganglia and tooth germs in their specific culture media (Park et al., 2006). At the same time microfluidics devices allow the spreading of axons from the ganglia toward the tooth germs through the microgrooves. To recapitulate the *in vivo* pattern of tooth innervation in these devices, trigeminal ganglia were co-cultured for 10 days with embryonic or postnatal molar tooth germs. When cultured alone in the microfluidic devices, trigeminal ganglia spread neurites toward the other compartment (Figures 3A,B).

### Co-culture of embryonic trigeminal ganglia and embryonic molar tooth germs

**Figure 4:**
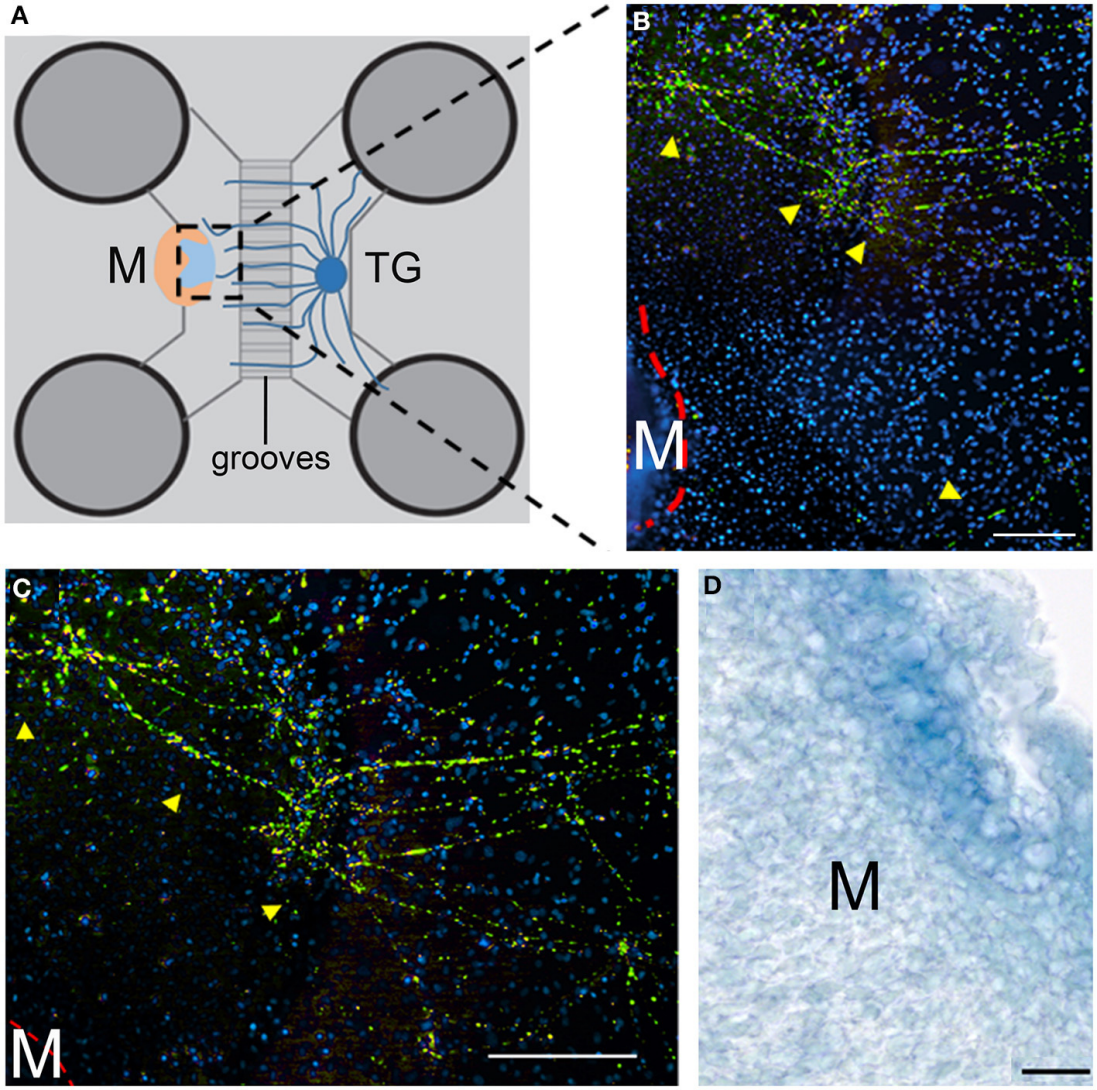
**Co-culture of trigeminal ganglia (TG) and embryonic molars in a microfluidic co-culture system. (A)** Schematic representation showing the co-culture of E15.5 trigeminal ganglia and E17.5 molars in the microfluidics chamber. The tooth is oriented with the pulp facing the ganglion. **(B)** Neurites (yellow arrows) grow toward the developing tooth (red line) but do not contact the tooth. Green: neurofilament; blue: DAPI. **(C)** Higher magnification of the upper area of panel **(B)**. Neurites are highlighted with yellow arrowheads. Green: neurofilament; blue: DAPI. **(D)** Section of a cultured tooth germ. Neurofilament immunostaining shows that nerve fibers did not enter into the E17.5 molars after 10 days of co-culture. Scale bars: 200 μm for **(B,C)** and 20 μm for **(D)**. TG, trigeminal ganglion; M, molar.

Axons from the trigeminal ganglion spread toward the E17.5 molar tooth germs and progressively surrounded them. Axons are visualized via immunostaining using an antibody against neurofilament (Figures 4A–C, green), a general axonal marker. In this co-culture system, as happened with the conventional system, the growing neurites did not approach or contact the dental tissues (Figures 4B,C). Axons grew through a layer of mesenchymal cells (spreading from the external layers of the tooth) without entering into the dental papilla (Figure 4C).

### Localization of nerve fibers in cultured embryonic molar tooth germs

To confirm that nerve fibers have not penetrated into the cultured embryonic tooth germs, the latter were removed from the culture dish and sectioned. We then performed immunohistochemistry using an antibody against neurofilament. As expected, no staining was detected in sections of the embryonic molar tooth germs after 10 days of culture (Figure 4D). This confirms that axons did not penetrate into the tooth germs; similarly to *in vivo* (Mohamed and Atkinson, 1983), axons from the trigeminal ganglion approached and tended to surround the tooth germ, but they do not contact it until later stages of development.

### Co-cultures of embryonic trigeminal ganglia and postnatal molar tooth germs

**Figure 5:**
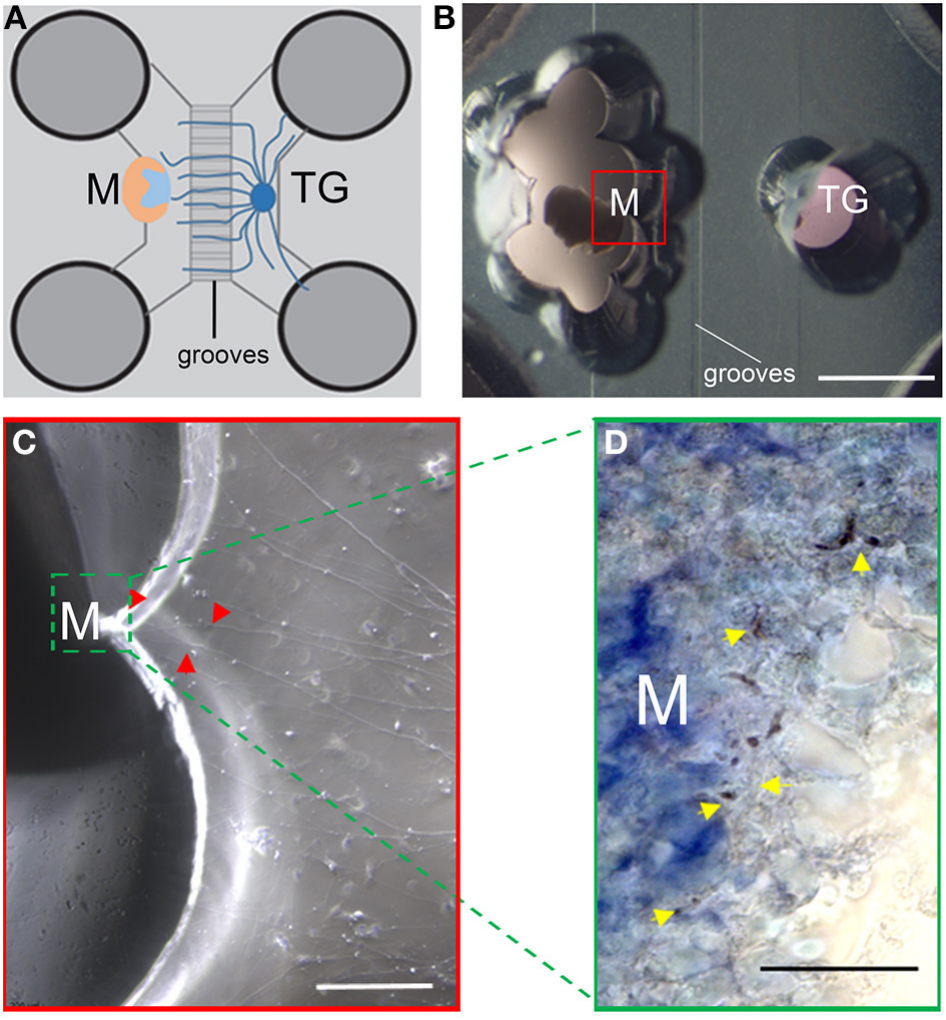
**Co-culture of trigeminal ganglia (TG) and postnatal molars**. **(A)** Schematic representation showing the disposition of E15.5 TG and postnatal molars in the microfluidics chamber. The molar is oriented with the pulp facing the ganglion. **(B)** PN5 molar (on the left) and E15.5 TG after 10 days of culture. Red box: magnification in panel **(C)**. **(C)** Neurites (red arrowheads) grow toward the tooth germ (green line) and penetrate into the tooth germ. **(D)** Section of the cultured tooth germ. Neurofilament staining shows that neurites have contacted the dental pulp (yellow arrowheads). Scale bars: 2 mm for **(B)**, 200 μm for **(C)**, and 20 μm for **(D)**. Abbreviations: TG, trigeminal ganglion; M, molar.

In the microfludics co-cultures (Figures 5A,B), nerve fibers from the trigeminal ganglion grew toward the postnatal molar tooth germs without showing any sign of repulsion (Figure 5C). In contrast to the previous set of experiments, neurons entered into the dental pulp mesenchyme after the culture period (Figure 5C). Molars preserved their structure in these culture conditions (Figure 5B).

### Localization of nerve fibers in cultured postnatal molar tooth germs

To confirm the above results, we performed immunostaining using the neurofilament antibody on sections of the cultured tooth. Neurofilament staining clearly marked nerve fibers in the pulp of postnatal molar tooth germs after 10 days of culture (Figure 5D). This confirms that nerve fibers from the trigeminal ganglion innervate postnatal molars, as observed *in vivo* (Mohamed and Atkinson, 1983).

## Discussion

Recent studies suggest that innervation is pivotal during embryonic development and regeneration of many tissues and organs (Pagella et al., 2014). For example, in the orofacial complex, it has been demonstrated that innervation plays an active role in the morphogenesis of salivary glands (Knox et al., 2010, 2013) and taste buds (Hosley et al., 1987; Oakley et al., 1998; Liebl et al., 1999; Mistretta et al., 1999; Oakley and Witt, 2004; Guagliardo and Hill, 2007). Furthermore, it has been shown that innervation regulates stem cell activation and differentiation of various organs (Katayama et al., 2006; Brownell et al., 2011; Fitch et al., 2012), including mouse incisors (Zhao et al., 2014). Although a considerable effort has been made during the last decades to understand the molecular mechanisms underlying the interactions between axons and their target tissues, the role of innervation in the development and regeneration of orofacial organs, and particularly in teeth, is still neglected and controversial.

Tooth innervation is a spatiotemporally controlled process, tightly linked with the development of dental tissues (Hildebrand et al., 1995; Luukko and Kettunen, 2014). Pioneer trigeminal axons project toward the tooth germs from the earliest stages of their development (Stainier and Gilbert, 1990). Axons follow specific pathways that are determined by the expression of defined proteins (Kettunen et al., 2005). Nerve fibers surround the developing tooth germs, but they do not enter the dental papilla mesenchyme until the first deposition of minerals and formation of dentin and enamel (Mitsiadis and Luukko, 1995; Luukko, 1997; Moe et al., 2008). Innervation is regulated by a plethora of molecules that are expressed by tooth germs such as neurotrophins (Mitsiadis et al., 1992, 1993; Mitsiadis and Luukko, 1995), semaphorins (Kettunen et al., 2005), ephrins, netrins, cell-adhesion molecules and laminins (Luukko and Kettunen, 2014; Pagella et al., 2014). The expression pattern of these factors is dynamic and spatiotemporally regulated during odontogenesis. Upregulation of NGF and BDNF expression in odontoblasts is associated with both dentin formation and dental pulp innervation, suggesting that neurotrophins are important molecules for axonal attraction into the dental pulp (Kettunen et al., 2005; Moe et al., 2012). Consistently, deletion of NGF signaling in tooth leads to decreased pulp innervation (Matsuo et al., 2001). In contrast, semaphorins, and in particular semaphorin 3A, exert a repulsive effect on sensory neurons. In dental mesenchyme, semaphorins show a strictly spatio-temporal expression pattern that may guide neurite growth during the different stages of odontogenesis (Kettunen et al., 2005; Moe et al., 2012; Luukko and Kettunen, 2014). These results provide significant information about the effects of the various molecules involved in axon attraction. However, in order to unravel the different molecular players in interactions between neurons and their target tissues, *in vitro* co-culture approaches, in combination with *in vivo* genetic manipulations, are necessary. *In vitro* co-culture approaches allow studying these interactions in isolated and controlled systems that permit precise manipulations at the molecular level.

Previous studies were based on conventional co-cultures of trigeminal ganglia and tissues or cells of the orofacial area (Lillesaar et al., 1999, 2001; Nosrat et al., 2001; Lillesaar and Fried, 2004; Wyatt et al., 2011). These studies were conducted to investigate mainly the attractive effects of these cells or tissues on sensory axons (O'Connor and Tessier-Lavigne, 1999). Although bringing significant advances in the field, several technical issues were raised. Studies realized with conventional culture conditions with rat tissues have shown that either postnatal dental pulp explants or pulp cells elicit neurites growth from trigeminal ganglia (Lillesaar et al., 1999, 2001). Dental explants from various developmental stages have provoked different responses in trigeminal neurons (Lillesaar and Fried, 2004). We showed here that embryonic incisors co-cultured with trigeminal ganglia in the same medium maintain their repulsive effect on neurites. These results confirm previous observations (Lillesaar and Fried, 2004) and are representative of the *in vivo* situation. However, tooth germs start to degenerate after few days of culture in these conditions. In the majority of the cases, previous conventional co-cultures were performed for short periods of time that were generally not longer than two days (Lillesaar et al., 1999, 2001; O'Connor and Tessier-Lavigne, 1999; Lillesaar and Fried, 2004). In fact, dental tissues and trigeminal ganglia require different culture conditions (Mitsiadis and Drouin, 2008; Petrinovic et al., 2010; Otsu et al., 2012). Growing neurons and teeth in the same culture conditions will impair any eventual analysis of molecules involved in the cross talk between these two tissues. Optimal culture conditions are needed to preserve the physiological molecular profile of trigeminal ganglia and dental tissues. Conventional co-cultures have been applied previously to investigate the innervation-dependent secretion of neurotrophins by maxillary tissues (Wyatt et al., 2011). However, in these studies, trigeminal ganglia and maxillary processes have been exposed to the same culture conditions. Moreover, co-cultures were maintained for a short period of time that is insufficient for studying the influence of innervation in maxillary development.

Microfluidics systems have been used so far to co-culture neurons and various cell types in optimized media (Hosmane et al., 2012). This microfluidics system can represent more faithfully the *in vivo* situation, where neural cell bodies, axonal terminals and target tissues are generally exposed to different cellular and molecular microenvironments. More recently, microfluidics devices have been used to co-culture whole dorsal root ganglia and osteoblasts (Neto et al., 2014). Indeed neurites from dorsal root ganglia grew toward the compartment containing the osteoblast in the absence of exogenous neurotrophins and formed functional synapses with their target cells (Neto et al., 2014). Based on these findings and considerations, microfluidics devises were used to co-culture embryonic trigeminal ganglia and developing tooth germs. Organotypic cultures have the clear advantage of maintaining the original structure of ganglia and teeth that is obviously lost in dissociated cultures. Trigeminal ganglia and embryonic or postnatal tooth germs co-cultured in microfluidic devices survive and grow properly for long periods (i.e., 10 days). In these co-cultures, embryonic tooth germs are surrounded by the growing neurons, but the neurons do not enter into the dental papilla, mimicking thus the *in vivo* situation (Mohamed and Atkinson, 1983). Conversely, neurons from the trigeminal ganglia innervate the pulp of postnatal teeth, which is also in accordance with the *in vivo* situation (Mohamed and Atkinson, 1983). In these microfluidics conditions tooth germs maintain their repealing or attracting effects on neurons and do not display any sign of tissue degeneration. Therefore, microfluidics systems could represent a proper platform allowing longer culture periods for the study of interactions between neurons and growing teeth. Moreover, separation of the neuronal from the dental compartment permits analysis of the effects of specific protein localization and quantification (Park et al., 2006). Blocking antibodies or recombinant proteins could also be added to the separate compartments of the microfluidics devices for analyzing their effects on neuronal and dental tissues. For example, treatment of trigeminal ganglia with NGF supports their survival and allows neuronal outgrowth. However, in this work exogenous NGF is absent from the tooth germ compartment, where tooth-derived signals are the only responsible for neuronal attraction or repulsion. Similarly, other recombinant molecules, antibodies or drugs can be used to manipulate the system in controlled conditions (Park et al., 2006).

In conclusion, microfluidics co-culture systems are optimal for investigating the role of innervation in developing or regenerating teeth and permit the study of interactions between neuronal and dental tissues.

### Conflict of interest statement

The authors declare that the research was conducted in the absence of any commercial or financial relationships that could be construed as a potential conflict of interest.

## Supplementary material

Supplementary Figure 1 **Comparison of E15.5 incisor cultured in optimal conditions and in conditions optimized for trigeminal ganglia cultures. (A)** E15.5 incisor cultured in tooth culture medium (see Materials and Methods) for 10 days. The tooth shape is elongated, and the limits of the tooth are clearly visible and defined. **(B)** E15.5 incisor co-cultured with a trigeminal ganglion in trigeminal ganglion medium (see Materials and Methods, magnification of Figure 2C). The incisor is smaller, and cells migrate massively from the tooth, particularly on the lingual (upper, red arrow) side. Scale bar: 500 μm.
