# Supplementary figures and images for: Microfluidics co-culture systems for studying tooth innervation
Source: Front Physiol. 2014 Aug 25;5:326. doi: 10.3389/fphys.2014.00326 (PMC4142415; doi:10.3389/fphys.2014.00326)

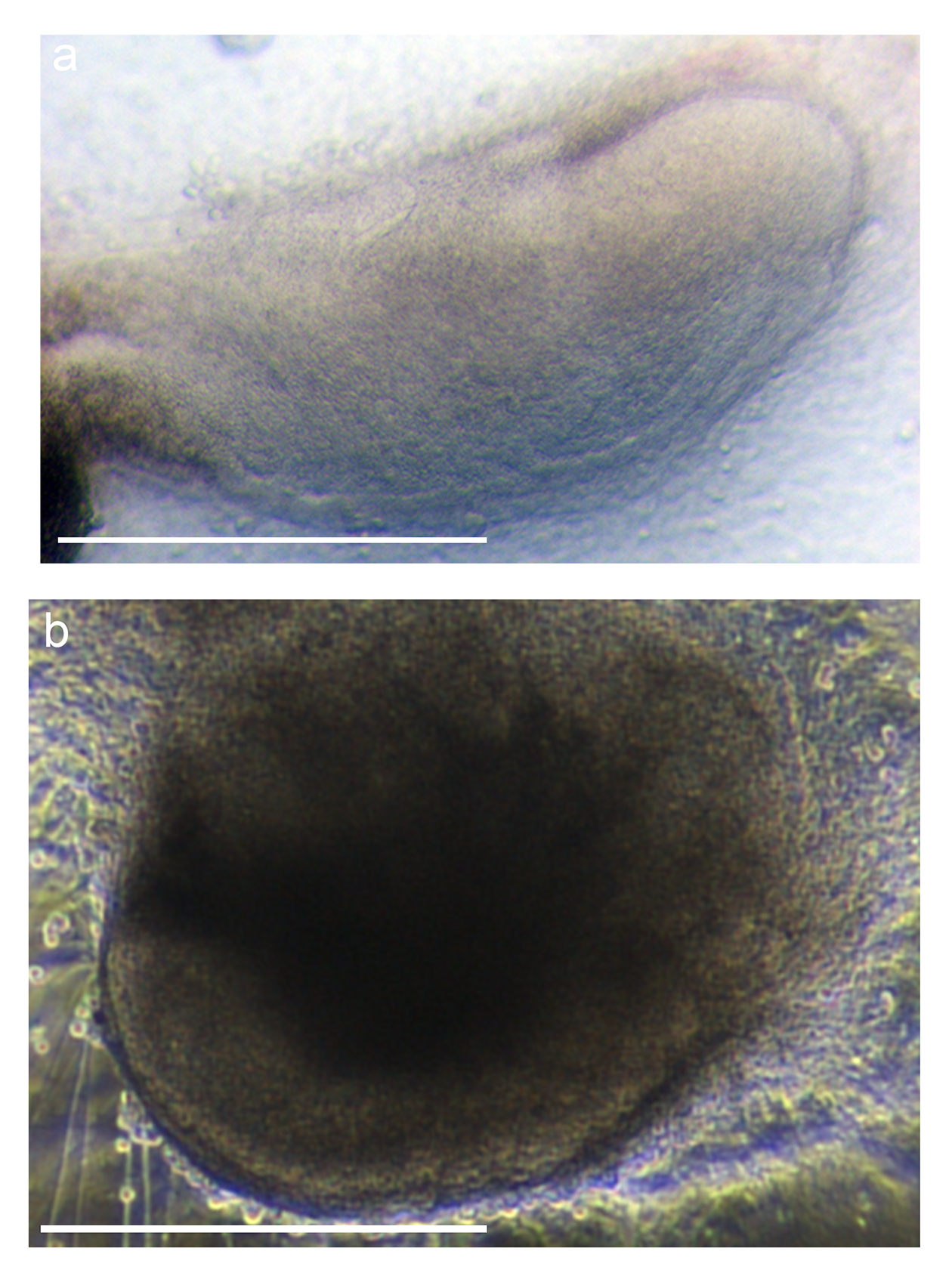

Supplement: Supplementary Figure 1 — Comparison of E15.5 incisor cultured in optimal conditions and in conditions optimized for trigeminal ganglia cultures. (A) E15.5 incisor cultured in tooth culture medium (see Materials and Methods) for 10 days. The tooth shape is elongated, and the limits of the tooth are clearly visible and defined. (B) E15.5 incisor co-cultured with a trigeminal ganglion in trigeminal ganglion medium (see Materials and Methods, magnification of Figure 2C). The incisor is smaller, and cells migrate massively from the tooth, particularly on the lingual (upper, red arrow) side. Scale bar: 500 μm. [file Image1.TIF]
